# Scrotal kidney: a gross case of “nephroptosis”

**DOI:** 10.1259/bjrcr.20220054

**Published:** 2023-03-08

**Authors:** Elmuiz A. Hsabo, Ikenna Anderson Aneke, Jonathan Tuck, Seshikanth Middela

**Affiliations:** 1 Department of Urology, Wythenshawe hospital, Manchester University NHS Foundation Trust, Manchester, United Kingdom; 2 Wythenshawe Hospital and The Christie, Manchester University NHS Foundation Trust, Manchester, United Kingdom; 3 Department of Radiology, Wythenshawe hospital, Manchester University NHS Foundation Trust, Manchester, United Kingdom

## Abstract

We radiologically illustrate a case of nephroptosis in an 82-year-old male whose right kidney had progressively descended into the right hemiscrotum. This was detected upon a recent visit to the accident and emergency department (A&E) where a computed tomography (CT) scan demonstrated the right kidney within the scrotum with a degree of hydronephrosis yet a stable renal function. The patient was managed conservatively as per the multidisciplinary team (MDT) meeting advice.

## Background

Nephroptosis, also known as “wandering” or “floating” kidney, is a condition where the kidney descends by more than 5 cm (or two vertebral bodies) when moving from supine to upright position.^1,2^ While the true incidence of this condition is still unknown, intravenous urogram (IVU) studies showed slight female preponderance as compared to males.^1^ Furthermore, it has been postulated that the lack of adequate perinephric support might be a key factor contributing to this excessive range of kidney mobility.^1^

Despite many affected individuals being entirely asymptomatic, the commonest symptom quoted in the literature appears to be abdominal pain which is likely the result of a combination of arterial ischemia, venous congestion and hydronephrosis triggered by mechanical effects on hilar structures.^3^ Other symptoms such as hypertension, nausea, vomiting, palpable abdominal mass or transient haematuria have also been reported.^1^ Nephroptosis, however, is considered a diagnosis of exclusion and a literature review suggests that imaging modalities like doppler-ultrasound scan, intravenous pyelogram and radionuclide scans can help clench the diagnosis.^1^

Here, we radiologically illustrate a right kidney that had progressively descended over the years to eventually settle in the right hemiscrotum. To the best of our knowledge, this is the first case ever where a nephroptotic kidney has reached below the level of the inguinal ligament.

## Case presentation

An 82-year-old man presented to A&E with lower abdominal pain and scrotal discomfort. Physical examination revealed a soft, non-tender abdomen. A large, slightly tender, irreducible right-sided inguinoscrotal hernia was noted along with a long-term urethral catheter from a previous episode of urinary retention followed by multiple failed trials without catheter (TWOC). Despite that, his vital signs were all normal. His renal function test showed a stable eGFR of 43 mL/min/1.73 m^2^ which was within the limits of his normal baseline, but the rest of his blood tests were unremarkable.

The patient was on treatment for hypertension, Type 2 diabetes mellitus, atrial fibrillation and chronic obstructive airway disease. He was also known to have transient ischaemic attacks, abdominal aortic aneurysm and ischaemic heart disease. He was an ex-smoker with very poor exercise tolerance and overall reduced mobility. His body mass index (BMI) at the time of presentation was 27.3.

**Figure 1.**
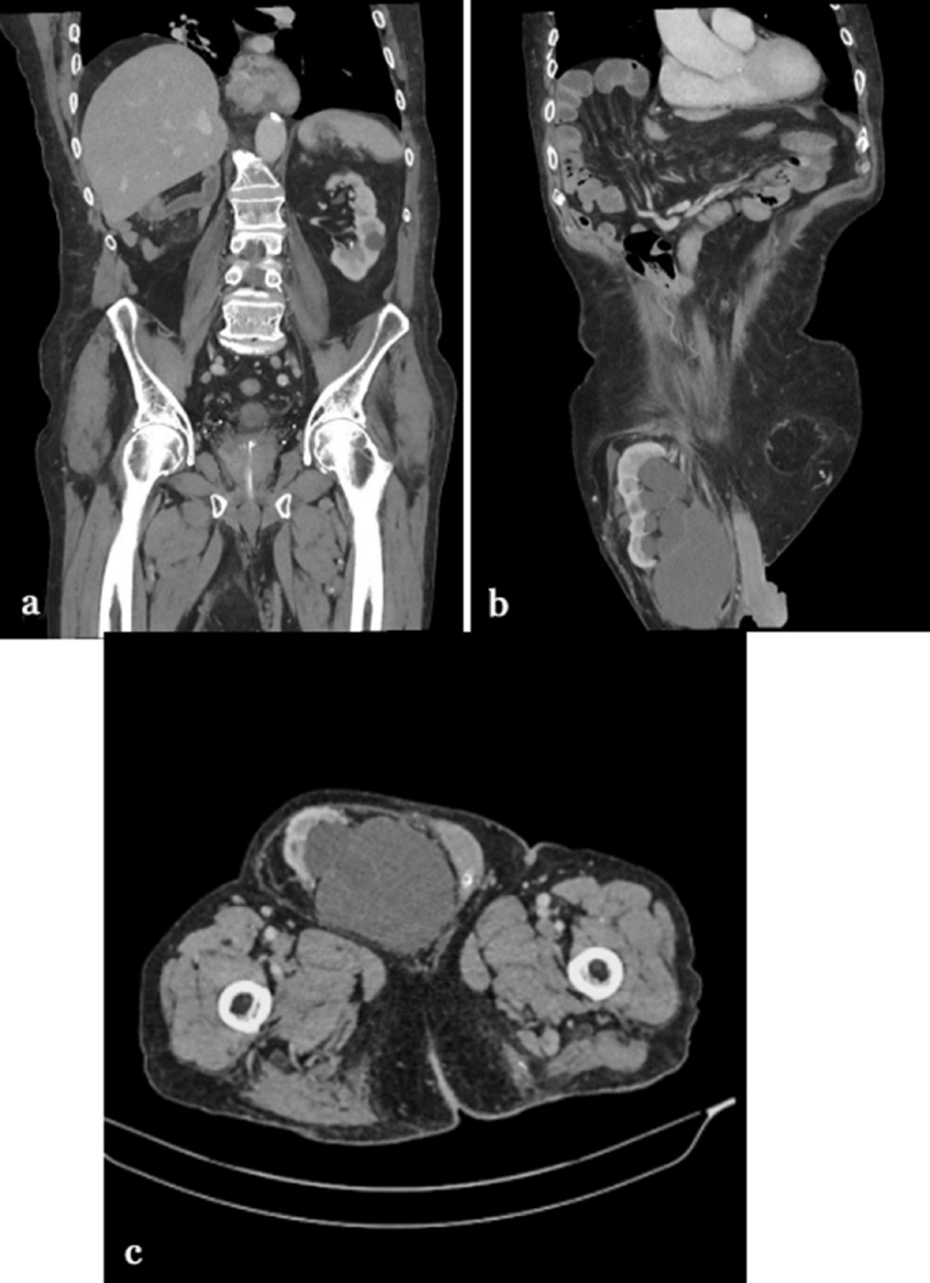
CT scan from 2021 showing (**a**) empty right renal fossa on a coronal slice (**b**) sagittal view demonstrating the right kidney in the right hemiscrotum with evidence of hydronephrosis along with moderate fluid in the hernial sac (**c**)the right kidney at the level of the proximal right femur.

Prior to this admission, the patient was followed up by the general surgery team for a large right-sided inguinoscrotal hernia, which was being managed non-operatively. Upon reviewing the images from 2019, the hernia was clearly shown (Figure 4). On this admission, a computed tomography of the abdomen and pelvis (CT) with contrast (Figure 1) was performed to rule out the possibility of strangulated hernia. This revealed none but confirmed the presence of a viable right kidney within the scrotal sac along with moderate hydro-nephrosis and extravasation of urine. The scan did not involve a delayed contrast phase as no urological pathology was suspected at the time of requesting the scan.

**Figure 2.**
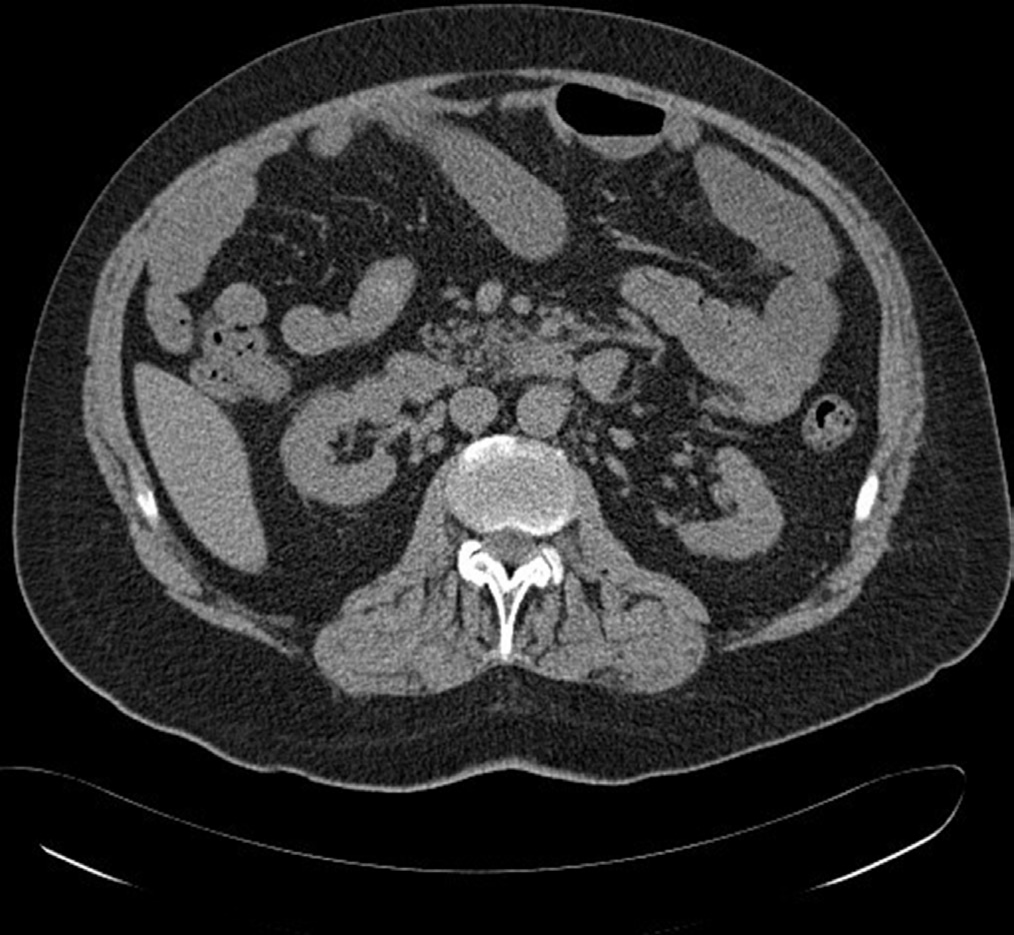
Axial view from a CT scan performed in 2008 showing the right kidney at the same level as the left kidney.

**Figure 3.**
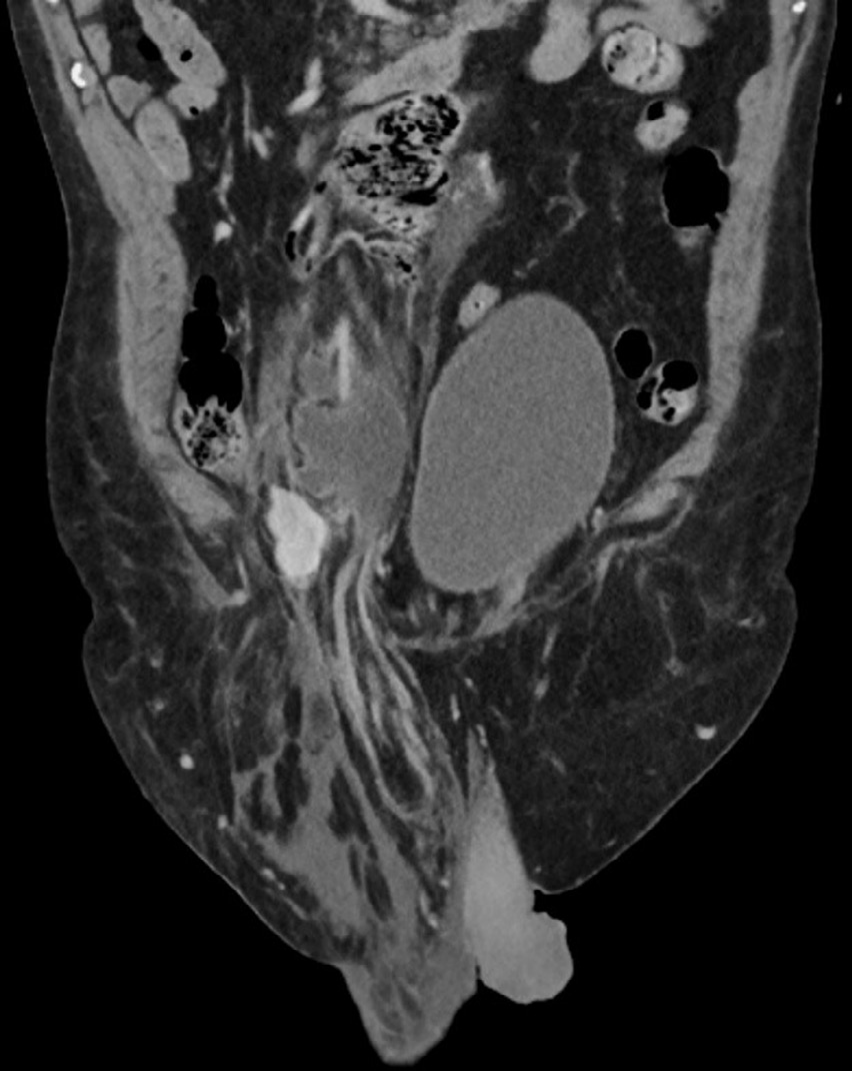
CT aortogram from 2019 showing the right ureter coiled within the hernial sac.

**Figure 4.**
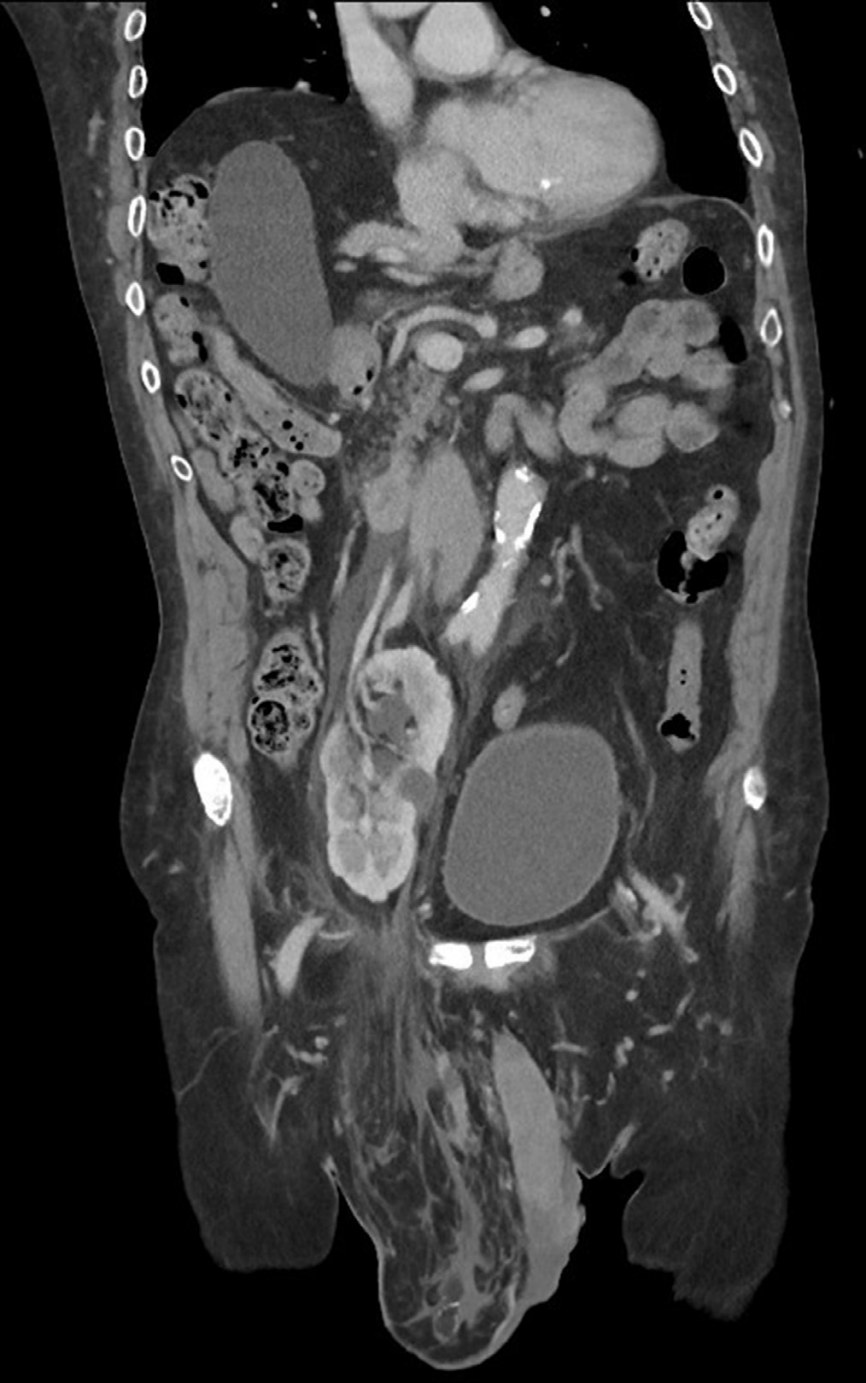
CT scan from 2019 depicting the large right inguinoscrotal hernia containing the tip of the lower pole of the right kidney with increased peri-nephric and hernial sac inflammatory changes as shown on the sagittal view.

To trace back the stages of kidney migration, we re-examined the patient’s previous radiological images that he underwent across different hospitals in our region over the past two decades. To begin with, the first CT chest and abdomen in 2008, which was arranged to exclude lung cancer, showed the right kidney parallel to the left kidney at L3 lumbar vertebral body level (Figure 2). Upon suspicion of acute intramural haematoma of the aorta in 2019, the patient underwent CT aortogram (Figure 3) where a considerable length of the right ureter was present in the hernial sac. Again, in the same year, another CT scan was performed to exclude bowel obstruction. This pointed out the right kidney in the right iliac fossa with its lower pole entering the inguinal hernia (Figure 4).

## Outcome and follow-up

This case was discussed at the urology MDT meeting due to its complexity. A conservative approach was advocated in view of the patient’s overall frailty, improved minimal symptoms and stable renal function with a reactive approach in the future should he become symptomatic. At the time of writing this case report, he continues to lead a relatively normal life with no further hospital admissions.

## Discussion

The term “nephroptosis” was first used in 1885^4^ and it was purely a diagnosis of exclusion after ruling out other causes of abdominal pain.^2^ In this regard, the accuracy of diagnosing nephroptosis in the pre-imaging era is questionable given the fact that the first ever attempt to visualize the upper urinary tract using contrast material took place in 1905 and it was not until 1930s that IVU became available for public use.^5^ Furthermore, the condition itself signifies a degree of kidney hypermobility, but whether this represents the extreme of a normal anatomical variant is yet to be explored.

Before the adoption of nephropexy, nephrectomy had long been considered the standard treatment for nephroptosis but was discontinued due to its excessive mortality rate.^1,2^ After being popularised by Hahn in 1881, it is interesting to note that nephropexy fell out of fashion in the late twentieth century mainly due to persistence of symptoms postoperatively.^2^ However, there seems to have been a surge in articles mentioning nephropexy in the literature over the past two decades due to the increased uptake of radionucleotide scans and the evolution of minimally invasive surgery.^1,2^

In this patient, the kidney appears to have slowly migrated caudally over the years remaining symptomless until it finally became lodged within the scrotal sac causing symptoms. This has resulted in significant elongation and stretching of its supplying vessels although this elongation does not seem to have resulted in any ischemic insult as one might expect. Moreover, it is reasonable here to assume that gravitational force exerts constant downward pull on the kidney, suspended by means of its hilar vessels, and this -in turn- causes the vessels to stretch even further. This effect might be potentiated by intrinsic soft tissue laxity in the perinephric and retroperitoneal region.

## Learning points

- The report captures an interesting and unique pictorial case.
- The risks versus benefits of any intervention must be discussed with the patient against their background performance status.
- Complex cases need multidisciplinary approach.
